# Clinical and Functional Improvement with Brexpiprazole in an Adolescent with Early-Onset Schizophrenis

**DOI:** 10.1192/j.eurpsy.2026.11133

**Published:** 2026-07-31

**Authors:** I. Martín, S. Pérez Sánchez, S. Pérez Sánchez

**Affiliations:** 1 Psychiatry; 2 Los Arcos Universitary Hospital, Murcia, Spain

## Abstract

**Introduction:**

Over the last few decades, the treatment of schizophrenia has evolved from a symptom-centered approach to one that emphasizes functional recovery and quality of life. In early-onset schizophrenia, where the illness interferes with key developmental processes, it is essential to select antipsychotic agents that effectively control symptoms while minimizing side effects and preserving cognitive and affective capacities.

**Objectives:**

To evaluate clinical evolution, functional improvement, and quality of life in an adolescent with early-onset schizophrenia treated with brexpiprazole.

**Methods:**

Clinical case presentation including symptom evolution, pharmacological strategy, and psychosocial interventions. Parental informed consent was obtained.

**Results:**

A 15-year-old female presented with progressive social and family withdrawal, mistrust of friends, and experiences of depersonalization and derealization. The first psychotic episode included severe anxiety, thought disorganization, persecutory and control delusions, auditory hallucinations, and behavioral disturbance culminating in a fire. She was initially stabilized with aripiprazole and lorazepam. Aripiprazole was maintained at 15 mg/day with adjunctive psychotherapy, leading to partial remission. After 12 months, akathisia and emotional blunting appeared, jeopardizing adherence. Dose reduction and benzodiazepine co-treatment led to relapse with re-emergence of hallucinations and disorganization. A gradual switch to brexpiprazole resulted in full stabilization and disappearance of akathisia. Brexpiprazole was maintained at 2.5 mg/day with good tolerability and adherence.

**Results:**

Psychotic symptoms remitted within 8 weeks on aripiprazole and within 6 weeks on brexpiprazole. The latter showed a more favorable impact on subjective well-being, perceived functionality, and cognitive performance. A comprehensive treatment plan combining pharmacotherapy, individual, family, and group therapy was implemented.

**Conclusions:**

Brexpiprazole appears to be a useful alternative for early-onset schizophrenia, offering effective symptom control with lower sedation and reduced risk of akathisia. Its tolerability may contribute to better adherence and preservation of cognitive and emotional capacities, thus improving the adolescent’s overall quality of life.

**Keywords:**

brexpiprazole; early-onset schizophrenia; quality of life1

**Disclosure of Interest:**

None Declared

